# Can addition of frozen section analysis to preoperative endometrial biopsy and MRI improve identification of high-risk endometrial cancer patients?

**DOI:** 10.1186/s12885-021-08910-5

**Published:** 2021-11-04

**Authors:** Go Nakai, Yoshikazu Tanaka, Takashi Yamada, Masahide Ohmichi, Kazuhiro Yamamoto, Keigo Osuga

**Affiliations:** 1 The department of diagnostic radiology, Osaka Medical and Pharmaceutical University, 2-7 Daigaku-machi, Takatsuki City, Osaka 569-8686 Japan; 2 The department of diagnostic radiology, Tesseikai Neurosurgical Hospital, Shijonawate City, Osaka Japan; 3 The department of pathology, Osaka Medical and Pharmaceutical University, Takatsuki City, Osaka Japan; 4 The department of Obstetrics and Gynecology, Osaka Medical and Pharmaceutical University, Takatsuki City, Osaka Japan

**Keywords:** Endometrial cancer, Frozen section, MRI, Preoperative endometrial biopsy

## Abstract

**Background:**

Surgeons sometimes have difficulty determining which result to favor when preoperative results (MRI + preoperative endometrial biopsy [pre-op EB]) differ from intraoperative frozen section histology (FS) results. Investigation of how FS can complement ordinary preoperative examinations like MRI and pre-op EB in identification of patients at high risk of lymph node metastasis (high-risk patients) could provide clarity on this issue. Therefore, the aim of this study is to assess the utility of pre-op EB, MRI and FS results and determine how to combine these results in identification of high-risk patients.

**Methods:**

The subjects were 172 patients with endometrial cancer. Patients with a histological high-grade tumor (HGT), namely, grade 3 endometrioid cancer, clear cell carcinoma or serous cell carcinoma, or with any type of cancer invading at least half of the uterine myometrium were considered high-risk. Tumors invading at least half of the uterine myometrium were classified as high-stage tumors (HST). We compared *(a)* detection of HGT using pre-op EB versus FS, *(b)* detection of HST using MRI versus FS, and *(c)* identification of high-risk patients using MRI + pre-op EB versus FS. Lastly, we determined to what degree addition of FS results improves identification of high-risk patients by routine MRI + pre-op EB.

**Results:**

*(a)* Sensitivity, specificity, and accuracy for detecting HGT were 59.6, 98.4 and 87.8% for pre-op EB versus 55.3, 99.2 and 87.2% for FS (*P* = 0.44). *(b)* These figures for detecting HST were 74.4, 83.0 and 80.8% for MRI versus 46.5, 99.2 and 86.0% for FS (*P* < 0.001). *(c)* These figures for identifying high-risk patients were 78.3, 85.4 and 82.6% for MRI + pre-op EB versus 55.1, 99.0 and 81.2% for FS (*P* < 0.001). The high specificity of FS improved the sensitivity of MRI + pre-op EB from 78.3 to 81.2%, but this difference was not statistically significant (*P* < 0.16).

**Conclusion:**

Frozen section enables identification of high-risk patients with nearly 100% specificity. This advantage can be used to improve sensitivity for identification of high-risk patients by routine MRI + pre-op EB, although this improvement is not statistically significant.

## Introduction

Addition of lymphadenectomy to surgery for endometrial cancer increases the risk of complications such as surgery-related systemic morbidity, lymphedema and lymphocele formation [1]. Current guidelines for the treatment of endometrial cancer suggest that lymphadenectomy can be omitted in low-risk patients (defined as those with grade 1 or 2 endometrioid adenocarcinoma without deep myometrial invasion [MI]) because recent randomized trials have shown that lymphadenectomy does not provide survival benefit in these patients [2–4]. Therefore, histologic grade and the extent of disease, including the depth of MI, are major factors used to differentiate patients at high risk of lymph node metastasis (high-risk patients) from low-risk patients. Histologic type and grade are determined by preoperative endometrial biopsy (pre-op EB), while preoperative magnetic resonance imaging (MRI) can assess the depth of MI as well as the extent of disease, including cervical involvement, peritoneal dissemination, and adnexal tumors [5]. Thus, both tests aid in preoperative stratification of patients into high- and low-risk groups. The American College of Obstetricians and Gynecologists 2005 Practice Bulletin recommended analysis of intraoperative frozen sections (FS) of the uterine tumor to minimize over- and under-treatment while the 1988 FIGO (International Federation of Gynecology and Obstetrics) staging system was still current [6]. However, not all hospitals can perform intra-operative FS and the accuracy of FS in predicting MI of endometrial cancer remains controversial [7, 8]. Furthermore, surgeons can have difficulty determining which result to favor when there is a discrepancy between preoperative (MRI + pre-op EB) and FS results. Therefore, the aim of this study is to assess the utility of pre-op EB, MRI and FS results and determine how to combine these results in identification of high-risk patients.

## Methods

### Study population

**Fig. 1:**
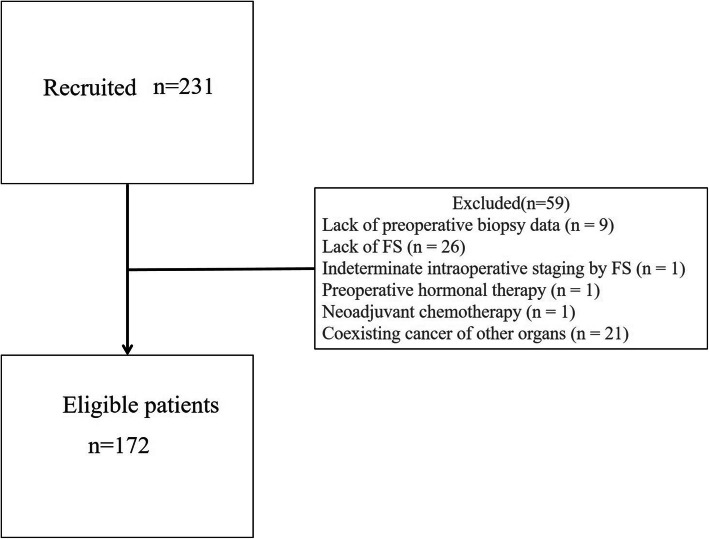
Flowchart shows study participant inclusion and exclusion. Consecutive patients who underwent surgery for uterine endometrial cancer between January 2013 and September 2016 at this hospital were recruited. Thirty-six patients were excluded due to incomplete preoperative assessment data, including lack of pre-op EB data (*n* = 9), lack of FS (*n* = 26), or indeterminate intraoperative staging by frozen section (*n* = 1). Patients who underwent preoperative hormonal therapy (*n* = 1) or neoadjuvant chemotherapy (*n* = 1), or had coexisting cancer of other organs, including uterine cervical cancer and non-endometrioid ovarian cancer (*n* = 21), were also excluded. FS: frozen section. Pre-op EB: preoperative endometrial biopsy

A total of 231 consecutive patients who underwent surgery for uterine endometrial cancer at our hospital between January 2013 and September 2016 were considered for the study. Thirty-six patients were excluded due to incomplete preoperative assessment data, including lack of pre-op EB data (*n* = 9), lack of FS (*n* = 26), or indeterminate intraoperative staging by FS (*n* = 1). Patients who underwent preoperative hormonal therapy (*n* = 1) or neoadjuvant chemotherapy (*n* = 1) or had coexisting cancer of other organs including uterine cervical cancer and non-endometrioid ovarian cancer (*n* = 21) were also excluded. Consequently, a total of 172 patients were included in the analysis (Fig. 1). This was a retrospective study conducted with the approval of the ethics committee of Osaka Medical Pharmaceutical University (No. 2736). Informed consent was obtained by allowing patients to opt out of the study on the hospital website and no patient declined to participate. All patients underwent preoperative MRI followed by total hysterectomy with bilateral adnexectomy and pelvic lymphadenectomy. The average interval between MRI and the operation was 45.1 days (range, 16–125 days). Para-aortic lymphadenectomy was also performed for 46 patients when the preoperative histological type was grade 3 endometrioid cancer, clear cell carcinoma or serous cell carcinoma, the clinical stage was Ib, II, or III, or peritoneal washing cytology was positive.

### Definitions of high-risk patient, high-grade tumor, and high-stage tumor

High-risk patients were defined as those with a histological high-grade tumor (HGT), namely, grade (G) 3 endometrioid cancer, clear cell carcinoma or serous cell carcinoma or with any type of cancer invading at least half of the uterine myometrium. A high-stage tumor (HST) was defined as a tumor invading at least half of the uterine myometrium.

Low-risk patients were defined as those with a histological low-grade tumor (LGT), namely, atypical endometrial hyperplasia (AEH) or G1 or G2 cancer invading less than half of the uterine myometrium. A low-stage tumor (LST) was defined as a tumor invading less than half of the uterine myometrium.

### Imaging protocol

Preoperative MRI studies using multi-phase array coil were performed with a 1.5- or 3.0-T magnet for all patients without using antiperistaltic agents. The scanning parameters for T2-weighted fast spin-echo sequences in sagittal, oblique axial (short-axis view of uterus), and axial planes were as follows: repetition time range/echo time range, 4000–6000/100; field-of-view, 200 to 360 mm; slice thickness, 4 mm, interslice gap 1 mm, matrix 256 × 192 to 224 × 384. Axial T1-weighted images (T1WIs) (500–650/9) were acquired for all patients. Diffusion-weighted images (DWIs) were acquired in the axial and/or sagittal plane using the single-shot echo-planar technique. The scanning parameters at b values of 0 and 800 or 1000 s/mm^2^ were as follows: 3400–5500/68; slice thickness, 3 mm; interslice gap, 0 mm; matrix, 128 × 192; field-of view, 36 cm; number of excitations, 4–7. Unenhanced and dynamic (waiting times: 35, 80, 130 s) gadoxetic acid–enhanced images were obtained using spectral presaturation with inversion recovery (SPAIR) fat-suppressed T1-weighted imaging in axial and/or axial oblique planes. A 0.01-mmol/kg body weight dose of contrast agent was administered intravenously using a power injector at a rate of 2.0 ml/s, followed by a 20-ml saline flush.

### Preoperative endometrial biopsy and MRI analysis

Preoperative histological examination of the endometrial tumor was performed by curettage with or without hysteroscopy.

Two radiologists (G.N. and Y.T., 14 and 8 years of experience in pelvic MR imaging, respectively) staged endometrial cancer by MRI retrospectively. Neither knew the previously determined stage for each patient at the time of their independent evaluation. A consensus reading was performed when there was a difference in opinion. Preoperative evaluation by MRI was conducted according to the 8th edition of the Union for International Cancer Control (UICC)-Tumor Node Metastasis (TNM) classification, and only the T factor was assessed by MRI. Endometrial cancer typically shows slightly higher signal intensity than the uterine myometrium on T2WI, high signal intensity on DWI, and weaker enhancement than the uterine myometrium on contrast-enhanced images. Tumors confined to the endometrium, as well as those invading the inner half of the myometrium, were classified as T1a, and those invading one-half or more of the myometrium as T1b tumors. The MRI sequence showing the highest contrast between the tumor and the myometrium was given the greatest weight in determination of MI. Tumors with cervical stromal invasion were classified as T2. Intraperitoneal nodules with similar signal intensity to endometrial cancer on MRI were regarded as invasion or dissemination. Tumors in the serosa or adnexa were classified as T3a, and those in the vagina or parametrium as T3b. Tumors directly invading the bladder or rectal mucosa were classified as stage T4a. Consequently, tumors classified as T1 correspond to LST and those greater than T1 correspond to HST on MRI.

### Preparation of intraoperative frozen sections from uterine tumors

Shortly after hysterectomy, the attending gynecologist visually determined the point of deepest penetration of the endometrial tumor into the uterine wall and selected this site as the representative site from which to prepare FS. Generally, one section was taken for FS analysis. The block was embedded in Tissue-Plus® O.C.T. (Optimal Cutting Temperature) Compound, frozen using liquid nitrogen, and then cut into slices at 4- to 6-μm intervals using a Leica CM1950 Cryostat Microtome. The slices were mounted on glass slides stained with hematoxylin and eosin, and evaluated microscopically for histologic type and the extent of MI.

### Postoperative histological analysis

The postoperative histology was considered as the reference standard. In this study, all ovarian endometrioid cancers were regarded as metastases because it is not always easy to determine whether the ovarian mass is primary or metastatic, especially when the endometrial cancer and ovarian cancer show similar histologic findings. Intraoperative FS histology and postoperative histology were assessed by one gynecologic pathologist (T.Y., 20 years of experience).

### Medical record data review and analysis

Clinical information including surgical records, pathology reports and tumor markers were recorded for all patients. The following were compared between patients classified as low- or high-risk based on postoperative histology: *(a)* detection of HGT using pre-op EB versus FS, *(b)* detection of HST using MRI versus FS, *(c)* identification of high-risk patients using MRI + pre-op EB versus FS. Lastly, we assessed to what degree addition of FS results improves the accuracy of routine MRI + pre-op EB for identification of high-risk patients.

### Statistical analysis

Statistical analyses were performed with JMP® 15.1.0 software (SAS Institute Inc., Cary, NC, USA). The Wilcoxon rank-sum test was used to compare age and tumor markers, and Fisher’s exact test was used to compare the number of low- and high-risk patients with lymph node metastasis. McNemar’s test was performed to compare the sensitivity and specificity of the two diagnostic methods and to evaluate concordance between the two methods, as well as to assess to what extent FS results improve the accuracy of routine MRI + pre-op EB for identification of high-risk patients. All *P* values less than 0.05 were considered significant.

## Results

The mean age of the patients was 56.5 ± 10.9 years (range, 28–84 years). Mean age differed significantly between patients classified as low-risk (*n* = 103, 54.3 years) and high-risk (*n* = 69, 59.8 years) by postoperative histology (*P* < 0.001). Histologic subtypes in low-risk patients included 2 atypical polypoid adenomyomas (APAM), 5 AEH, 71 G1, and 23 G2. A cancerous endometrial lesion was not detected postoperatively in 2 patients who had endometrial cancer diagnosed by pre-op EB. Histologic subtypes in high-risk patients were 18 G1, 4 G2, 23 G3, 4 clear cell carcinomas, 12 serous carcinomas, 6 carcinosarcomas, and 2 other subtypes. There were 13 high-risk patients with lymph node metastasis, but 0 low-risk patients (*P* < 0.001). CA125 and CEA differed significantly between patients classified as low- and high-risk based on postoperative histology with considerable overlap between them (37.7 ± 116.5 vs 139.2 ± 414.5 U/ml; *P* < 0.001 and 4.35 ± 26.4 vs 5.16 ± 15.0 ng/ml; *P* = 0.02, respectively). The characteristics of low- and high-risk patients are summarized in Table 1. *(a)* The respective sensitivity, specificity, positive predictive value (PPV), negative predictive value (NPV), and accuracy for detecting HGT were 59.6% (95% confidence interval [CI]: 0.45, 0.72), 98.4% (95% CI:0.94, 1.00), 93.3, 86.6 and 87.8% for pre-op EB versus 55.3% (95% CI: 0.41, 0.69), 99.2% (95% CI: 0.96, 1.00), 96.3, 85.5 and 87.2% for FS. The sensitivity and specificity of the two tests were not statistically different (*P* = 0.56 and 0.56 respectively). The difference between the two tests was not significant (*P* = 0.44). Results for evaluation of HGT are summarized in Table 2. Pre-op EB under-graded 19 tumors ultimately diagnosed as HGT. These consisted of 7 serous carcinomas (36.8%), 8 grade 3 endometrioid carcinomas (42.1%), 2 clear cell carcinomas (10.5%), 1 carcinosarcoma (5%), and 1 neuroendocrine tumor (5%). FS under-graded 21 tumors ultimately diagnosed as HGT. These consisted of 8 serous carcinomas (38.1%), 9 grade 3 endometrioid carcinomas (42.9%), 2 clear cell carcinomas (9.5%) and 1 carcinosarcoma (9.5%). Pre-op EB over-graded 2 tumors and FS over-graded 1 tumor ultimately diagnosed as LGT. All over-graded tumors were diagnosed as grade 3 but the diagnosis was changed to grade 2 on final pathology. Under- and over-grading of tumors by pre-op EB versus FS are summarized in Table 3. *(b)* The respective sensitivity, specificity, PPV, NPV, and accuracy for detecting HST were 74.4% (95% CI: 0.60, 0.85), 83.0% (95% CI: 0.75, 0.88), 59.3, 90.7 and 80.8% for MRI versus 46.5% (95% CI: 0.33, 0.61), 99.2% (95% CI: 0.96, 1.00), 95.2, 83.4 and 86.0% for FS. The sensitivity and specificity of the two tests were statistically different (*P* = 0.003 and *P* < 0.001 respectively). The difference between the two tests was significant (*P* < 0.001). Evaluation of HST is summarized in Table 2. Final whole specimen pathology showed that ovarian metastases were present in two cases. However, they were too small to be identified on MRI. MRI under-staged 13 tumors ultimately diagnosed as HST. These consisted of 6 Ib (46.2%), 5 IIIa (38.5%) and 2 IIIb (15.4%) cases. FS under-staged 25 tumors, which consisted of 11 Ib (44.0%), 1 II (4.0%), 9 IIIa (36.0%) and 4 IIIb (16.0%) cases. MRI over-staged 22 tumors and FS over-staged 1 tumor ultimately diagnosed as LST. All of the tumors over-staged by MRI and FS were staged as Ib. Under- and over-staging of tumors by MRI versus FS are summarized in Table 4. *(c)* The respective sensitivity, specificity, PPV, NPV, and accuracy for identification of high-risk patients were 78.3% (95% CI: 0.67, 0.86), 85.4% (95% CI: 0.77, 0.91), 78.3, 85.4, and 82.6% for MRI + pre-op EB versus 55.1% (95% CI: 0.43, 0.66), 99.0% (95% CI: 0.95, 1.00), 97.4, 76.7, and 81.2%. The sensitivity and specificity of the two tests were statistically different (*P* < 0.001 and *P* < 0.001, respectively). There was a significant difference between the two tests (*P* < 0.001).

**Table 1:** Characteristics of low-risk and high-risk patients

	Low-risk patients	High-risk patients	*P* value
N	103	69	
Age (y)	54.3 ± 9.53	59.8 ± 11.0	*P* < 0.001^b^
Histologic subtype	No lesion 2	G1 18	
	APAM 2	G2 4	
	AEH 5	G3 23	
	G1 71	CL 4	
	G2 23	S 12	
		CS 6	
		Others 2	
T factor (TNM classification)	T1a without myometrial invasion 23	T1a without myometrial invasion 3	
	T1a 80	T1a 22	
		T1b 22	
		T2 3	
		T3a 12	
		T3b 7	
Lymph node metastasis	0	13	*P* < 0.001^a^
CA125 (U/ml)	37.7 ± 116.5	139.2 ± 414.5	*P* < 0.001^b^
CEA (ng/ml)	4.35 ± 26.4	5.16 ± 15.0	0.02^b^
CA19–9 (U/ml)	55.1 ± 230.3	104.2 ± 255.3	0.74^b^

*APAM* atypical polypoid adenomyoma

*AEH* atypical endometrial hyperplasia

*G1, G2, G3* endometrioid adenocarcinoma grade 1, 2, 3

*CL* clear cell carcinoma

*S* serous carcinoma

*CS* carcinosarcoma

TNM classification: The 8th edition of the Union for International Cancer Control (UICC)-Tumor Node Metastasis (TNM) classification

^a^: Fisher’s exact test

^b^: Wilcoxon rank-sum test

**Table 2:** Diagnostic performance of preoperative examination (pre-op EB and/or MRI) versus frozen section for grade, stage, and risk level

High-grade tumor	Sensitivity	Specificity	PPV	NPV	Accuracy	*P* value
pre-op EB	59.6	98.4	93.3	86.6	87.8	0.44
Frozen sections	55.3	99.2	96.3	85.5	87.2	
*P* value	0.56	0.56				
High-stage tumor						
MRI	74.4	83.0	59.3	90.7	80.8	<0.001
FS	46.5	99.2	95.2	83.4	86.0	
*P* value	0.003	<0.001				
High-risk patient						
MRI + pre-op EB	78.3	85.4	78.3	85.4	82.6	<0.001
FS	55.1	99	97.4	76.7	81.2	
*P* value	<0.001	<0.001				
MRI + pre-op EB + FS	81.2	85.4	78.9	87.1	83.7	<0.16^a^
*P* value	0.16^a^	> 0.99^a^				

*pre-op EB* preoperative endometrial biopsy

*FS* frozen section

McNemar’s test was used for statistical analyses

^a^Versus MRI + pre-op EB

**Table 3:** Under- and over-grading of tumors by pre-op EB versus frozen section

Pre-op EB	Number (preoperative histology)	Postoperative histology	Number	%
Under-graded	19 (G1or G2)			
		Serous	7	36.8
		G3	8	42.1
		CL	2	10.5
		CS	1	5.3
		Others	1	5.3
Over-graded	2 (G3)			
		G2	2	100
Frozen section	Number (intraoperative histology)	Postoperative histology	Number	%
Under-graded	21 (G1or G2)			
		Serous	8	38.1
		G3	9	42.9
		CL	2	9.5
		CS	2	9.5
Over-graded	1 (G3)			
		G2	1	100

*G1, G2, G3* endometrioid adenocarcinoma grade 1, 2, 3

*CL* clear cell carcinoma

*S* serous carcinoma

*CS* carcinosarcoma

**Table 4:** Under- and over-staging of tumors by MRI versus frozen section

MRI	Number (preoperative stage)	Postoperative stage	Number	%
Under-staged	14 (Ia)			
		Ib	7	50
		IIIa	5	35.7
		IIIb	2	14.3
Over-staged	21 (Ib) 1 (IIIa)			
		Ia	22	100
Frozen section	Number (intraoperative histology)	Postoperative histology		
Under-staged	26 (Ia)			
		Ib	12	46.2
		II	1	3.8
		IIIa	9	34.6
		IIIb	4	15.4
Over-staged	1(Ib)			
		Ia	1	100

Given the extremely high specificity for identification of high-risk patients by FS, the FS result should be favored when it indicates high risk even if MRI + pre-op EB indicates low risk. When this approach was applied, two false negatives by MRI + pre-op EB turned to true positives by FS. Consequently, the sensitivity improved from 78.3 to 81.2%, but the change in sensitivity was not statistically significant (*P* = 0.16) and there was no significant difference between the two tests (*P* < 0.16) (Table 2).

## Discussion

In the initial management of endometrial cancer, it is important to distinguish patients who are at low risk of lymph node metastasis from those at intermediate to high risk to avoid overtreatment. Recently, sentinel lymph node (SLN) dissection has been recognized as standard practice in the management of patients with endometrial cancer [9]. However, intraoperative pathologic assessment of the primary tumor specimen may be used to determine the need for additional lymphadenectomy when SLN mapping has failed [10]. Therefore, even after the implementation of SLN mapping, preoperative evaluation of tumor grading and depth of myometrial invasion is still essential to predict risk of lymph node metastasis and guide treatment. Histologic grade and local stage, including MI, are strongly correlated with lymph node metastasis. Histologic grade is generally predicted by preoperative endometrial biopsy with or without hysteroscopy or intraoperative FS, while MRI is recommended for initial staging of endometrial cancer [5]. The diagnostic performance of DWI for detecting deep invasion of the myometrium is almost equal to that of intraoperative FS. Addition of DWI to conventional MR sequences (T2WI and dynamic T1WI) yielded the highest area under the ROC curve compared to FS or DWI (apparent diffusion coefficient map) alone [8]. Therefore, we included DWI in addition to T2WI and dynamic T1WI for local staging in this study. Sato et al. found that the sensitivity and specificity of MRI using only T2WI and dynamic T1WI for the diagnosis of greater than 50% MI depth were 75.0 and 85.7%, respectively [11]. In this study, we found that addition of DWI did not improve sensitivity or specificity, but this may have been because we did not use oblique axial DWI, which enables more accurate diagnosis of deep myometrial invasion than axial DWI [12].

Diagnostic accuracy for differentiation of HGT from LGT with pre-op EB and FS in this study were 87.8 and 87.2%. Additionally, sensitivity and specificity for differentiating HGT from LGT did not differ significantly between the two tests and the composition of histopathologic types in cases misdiagnosed (under- or over-graded) by pre-op EB was also quite similar to those misdiagnosed by FS. All three cases over-graded as G3 by pre-op EB or FS changed to G2 at final pathology. This is understandable because the histopathological difference between G2 and G3 depends on the number of tissue-forming glands relative to the total cancer volume, and thus the histopathological grade can change depending on the specific site of tissue collection by pre-op EB or FS. Sanjuan [13] and Ozturk [14] similarly reported respective accuracy figures of 89–91% and 87–90%, although overall concordance of grade between dilatation and curettage and the final pathological result is only 35.2–65.3% according to previous reports [7, 15]. Ugaki et al. reported no significant difference between the accuracy rate of diagnosis for histology on preoperative diagnosis and on FS diagnosis [16]. These findings are consistent with our results.

FS analysis has a risk of underdiagnosis of MI. A prospective study showed that diagnostic concordance with permanent specimens is as low as 67, and 28% (17/60) were upstaged by final histologic depth of invasion [17]. In this study, 9.3% of tumors classified as LST on MRI were upstaged to HST at final pathology, while 15.2% of those classified as LST on FS were upstaged. This result indicates that FS has a higher under-staging rate than MRI. The under-staging rate of FS previously reported in the literature ranges from 3.5 to 28% of all patients [7, 13, 14, 17]. We speculate that the discordance between FS and final pathology could be attributable to the site that the attending doctor selected for intraoperative FS analysis and that the selected specimen sliced at about 5 μm is not always representative for analysis of the deepest MI. The European Society of Gynaecological Oncology (ESGO) guidelines on uterine cancer do not recommend frozen section analysis because of its poor reproducibility and poor agreement with definitive paraffin sections [9]. We believe one reason for its poor reproducibility relates to bias in selection of specimens for frozen section. Our results may support the recommendation, considering that FS have lower sensitivity than MRI and pre-op EB and add no significant value to these tests. The fixation method may also affect diagnostic accuracy because frozen fixation cannot provide slides of as high a quality as formalin fixation without an appropriate fixation method. Furthermore, FS can only be used to evaluate MI intraoperatively, and not to assess lesions found in higher stages such as peritoneal dissemination, ovarian metastasis, parametrial involvement, and lymph node involvement. This contributes to a higher under-diagnosis rate than MRI, which can assess all lesions mentioned above preoperatively, even though lymph node involvement was not assessed by MRI in this study. We believe this may be why MRI could reduce underdiagnosed cases by almost half across all stages compared to FS in this study and showed statistically higher sensitivity for identification of HST and high-risk patients. On the other hand, other studies have similarly shown high specificity of greater than 93% for both tumor grade and MI by FS [13, 14, 18]. We assume that the significantly higher specificity for identification of high-risk patients by FS than by MRI + pre-op EB are due to its high specificity for both tumor grade and MI by FS. The extremely high specificity for identification of high-risk patients by FS indicate few false positive cases, and thus FS results indicating high risk should be favored to improve sensitivity for MRI + pre-op EB. This approach improved sensitivity from 78.3 to 81.2%, but the change was not statistically significant. We believe that this limited contribution is caused by the lower sensitivity and negative predictive value for identification of high-risk patients for FS than for MRI + pre-op EB (55.1 and 76.7% vs 87.3 and 85.4%, respectively).

This study has some limitations. First, this study involved loss of data and selection bias inherent to a retrospective study. Second, para-aortic lymphadenectomy was performed only for 46 patients, leading to verification bias. Third, lymphovascular space invasion (LVSI) was not included in the criteria for low-risk patients. When LVSI is divided into lymphatic vessel invasion (LVI) and blood vessel invasion (BVI), BVI is a strong independent prognostic factor for hematogenous metastases, while LVI is not [19]. Although peritumoral enhancement (PTE) on early dynamic contrast-enhanced images might correlate with both the presence and depth of MI and play an important role in the diagnosis of LVSI, the utility of PTE in detection of LVSI by MR findings seems low nonetheless (sensitivity, 43.4–47.6%; specificity, 81.5–83.3%; accuracy, 56.5–62.6%) [20]. Therefore, PTE was not evaluated in this study. However, Capozzi et al. found that prediction of lymphovascular space invasion in the primary tumor using the LVSI score may be useful to determine if lymphadenectomy should be performed when there is no mapping on a hemi-pelvis by the SLN technique [21]. Fourth, tumor volume was not assessed because the significance of association between pelvic and para-aortic lymph node metastases and tumor diameter is still controversial. Muallem et al. and Akbayir et al. reported there is no significant association between them when 2 cm is set as a cut-off value, although tumor volume is associated with tumor grade [22] and progression-free survival [23, 24]. Conversely, Todo et al. have validated the use of tumor volume in assessment of lymph node metastasis risk [25].

Lastly, differences in recurrence and survival rates between low- and high-risk patients were not assessed because our main endpoint was preoperative and intraoperative identification of high-risk patients. The Cancer Genome Atlas (TCGA) categorized endometrial cancer into four groups of carcinomas according to the somatic mutation pattern for the risk stratification of endometrial cancers adopted by the current WHO classification [26, 27]. When added to classic histological factors, these findings could produce a paradigm shift in predicting the prognosis of endometrial cancer.

## Conclusions

In conclusion, frozen section can identify high-risk patients with nearly 100% specificity. This advantage can be used to improve the sensitivity of routine MRI + pre-op EB for identification of high-risk patients, although this improvement is not statistically significant.

## Data Availability

The datasets used and/or analyzed during the current study are available from the corresponding author on reasonable request.

## Abbreviations

MRI: Magnetic resonance imaging
pre-op EB: preoperative endometrial biopsy
FS: Frozen section
HGT: High-grade tumor
LGT: Low-grade tumor
HST: High-stage tumors
LST: Low-stage tumors
MI: Myometrial invasion
FIGO: International Federation of Gynecology and Obstetrics
AEH: Atypical endometrial hyperplasia
G: Grade
WI: Weighted image
DWI: Diffusion-weighted image
SPAIR: Spectral presaturation with inversion recovery
UICC: the Union for International Cancer Control
TNM: Tumor Node Metastasis
O.C.T.: Optimal Cutting Temperature
CA: Cancer antigen
CEA: Carcinoembryonic antigen
PPV: Positive predictive value
NPV: Negative predictive value
CI: Confidence interval
LVSI: Lymphovascular space invasion
LVI: Lymphatic vessel invasion
BVI: Blood vessel invasion
PTE: Peritumoral enhancement
